# Confounder for Optic Disc Evaluation in Glaucoma

**DOI:** 10.7759/cureus.34621

**Published:** 2023-02-04

**Authors:** Archana R Thool, Kanchan V Selukar, Sachin V Daigavane

**Affiliations:** 1 Department of Ophthalmology, Jawaharlal Nehru Medical College, Datta Meghe Institute of Higher Education and Research, Wardha, IND

**Keywords:** field defect, optical coherence tomography, optic disc, angle closure glaucoma, disc coloboma

## Abstract

Congenital disc anomalies like optic disc coloboma or optic disc pit are rare occurrences. Coloboma involving disc or optic disc coloboma occurs due to defective closure of choroidal fissure, which can be unilateral or bilateral. These anomalies are discovered on routine examination or referred to as an open-angle glaucoma suspect. These anomalies can be asymptomatic or may present with visual field defects. Here we report a case of both eyes angle closure glaucoma with incidental finding of unilateral coloboma involving disc in the left eye. Optical coherence tomography of the optic nerve head showed peripapillary nerve fiber loss. Thus assessing such patients for diagnosis and the progression of visual field defects in managing glaucoma is quite challenging.

## Introduction

Glaucoma is a multifactorial disease characterized by raised intraocular pressure along with progressive retinal nerve fiber layer effect and optic disc cupping. Congenital disc anomalies like optic disc pit, optic nerve coloboma, morning glory syndrome, and tilted disc mimic glaucomatous optic disc damage morphologically. Also, these congenital anomalies may have nerve fiber layer loss and visual field defects [1]. The earliest sign of glaucomatous damage is a peripapillary nerve fiber layer defect seen up to five years before actual field loss [2]. Thus morphological appearance, nerve fiber layer defects, and visual field loss seen in patients having congenital disc anomalies present with a diagnostic dilemma and also the management of glaucoma. Usually, patients with coloboma of the disc or coloboma involving the disc are misdiagnosed as open angle glaucoma suspects [3]. Here we report a patient of recent onset angle closure glaucoma with incidental finding of unilateral coloboma involving the disc.

## Case presentation

**Figure 1:**
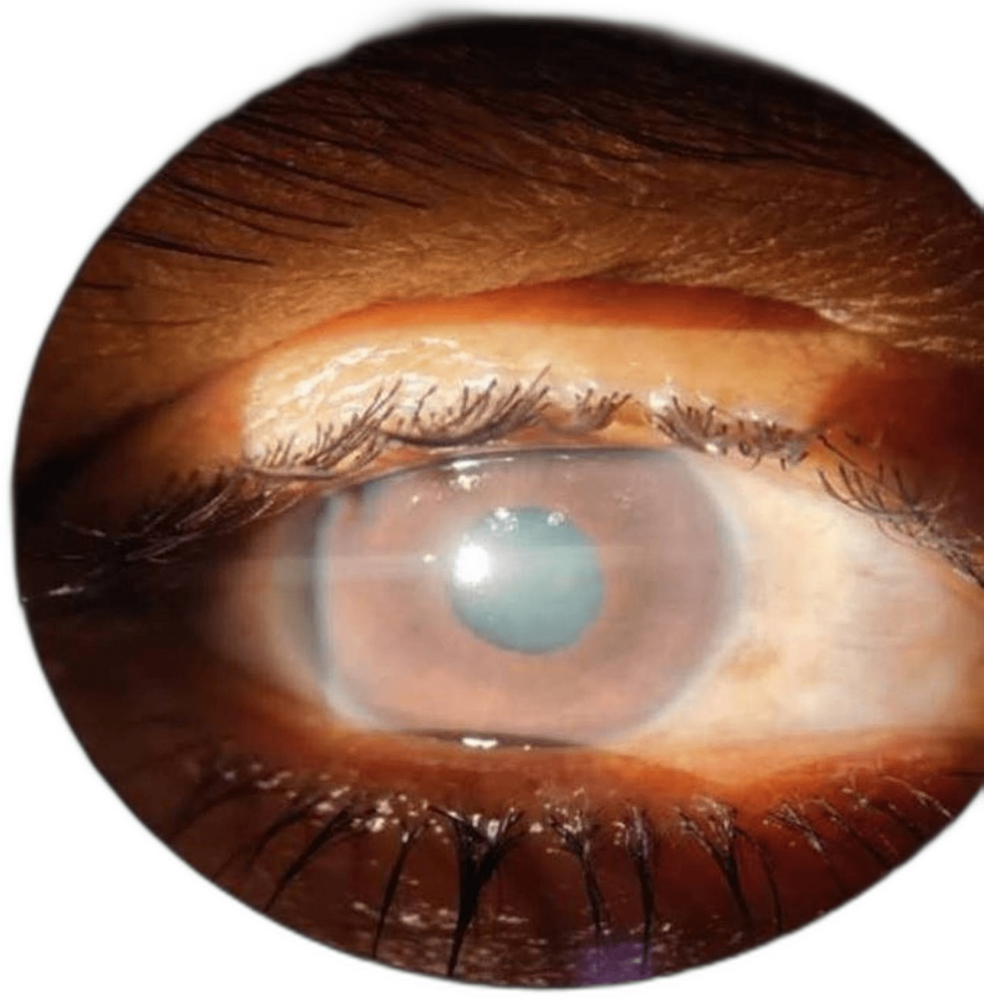
Slit lamp image of the right eye showing diffuse corneal edema

A 45-year-old female presented with an episode of acute angle closure glaucoma in her right eye for one week. There was a history of a similar episode in the left eye two months back. On examination, the right eye had diffuse corneal edema, mid-dilated non-reacting pupil, shallow anterior chamber, and clear lens (Figure 1).

**Figure 2:**
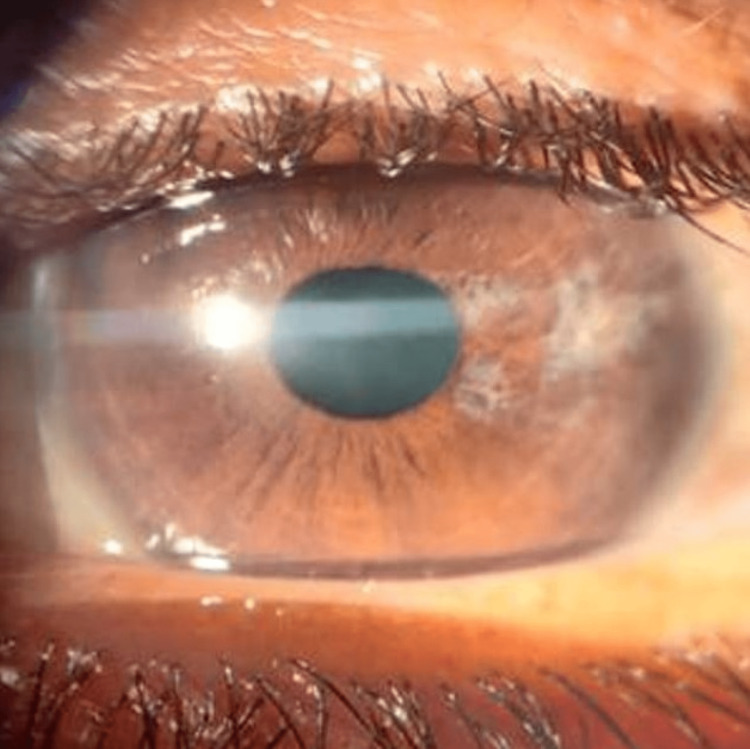
Slit lamp image of the left eye showing sectoral temporal iris atrophy

The left eye had a shallow anterior chamber and sectoral temporal iris atrophy (Figure 2).

**Figure 3:**
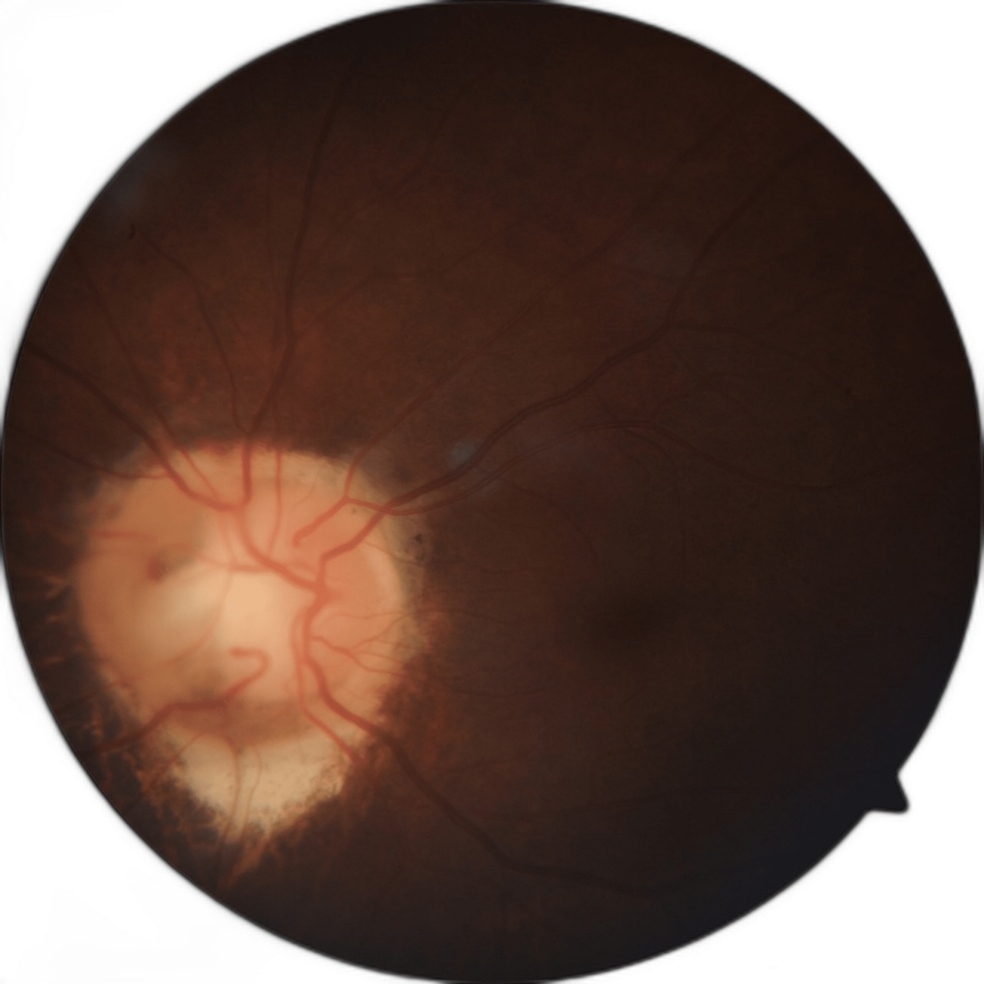
Fundus photo of the left eye showing coloboma involving optic disc

Intraocular pressure (IOP) measured by applanation tonometry was 56 mm Hg in the right eye and 16 mm Hg in the left eye. The acute episode was managed by intravenous 20% mannitol (1gm/Kg body weight) and topical antiglaucoma medication. After control of IOP, both eyes gonioscopy had a grade 1 angle. On fundus examination, the right eye had a normal optic disc with cup disc ratio of 0.3 and no slit or wedge defect in the peripapillary area. The peripheral fundus was normal. Whereas the left eye showed coloboma involving the optic disc (Figure 3), no anomalous vessels were noted. The peripheral fundus had no other colobomatous lesions.

**Figure 4:**
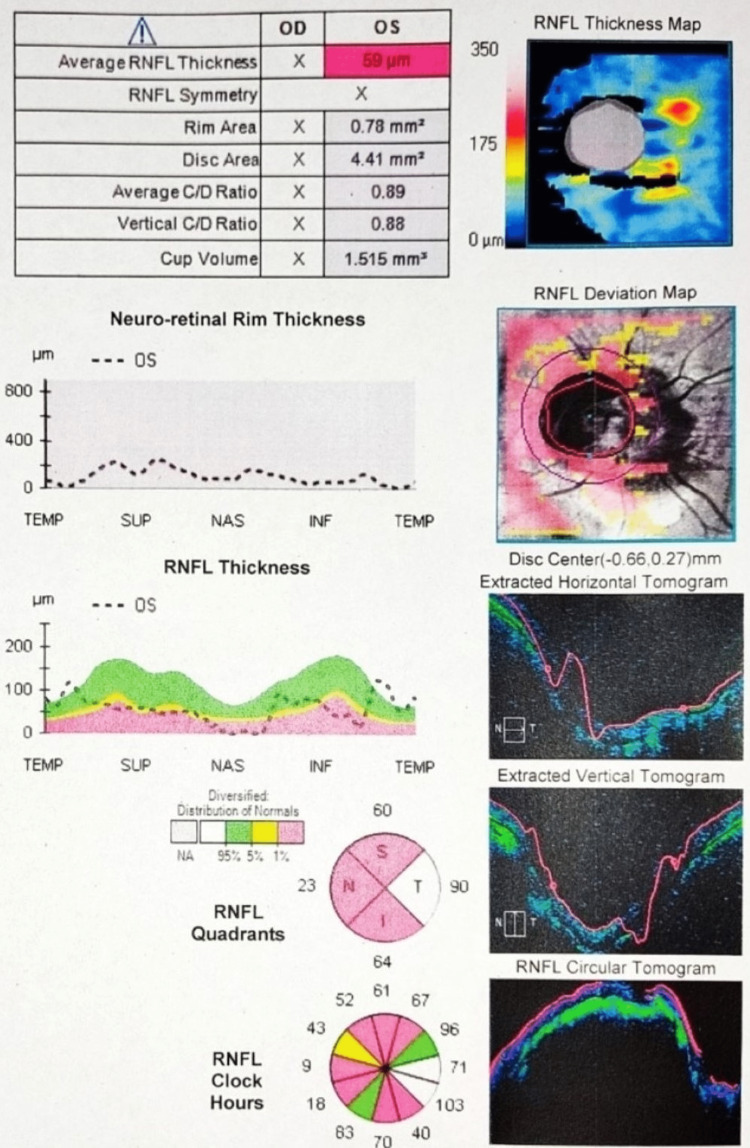
OCT ONH shows a decrease in nerve fiber thickness in the superior, inferior, and nasal quadrants OCT ONH - optical coherence tomography of the optic nerve head

Optical coherence tomography of the optic nerve head (OCT ONH) in the left eye showed peripapillary nerve fiber thickness decreased in the superior, inferior, and nasal quadrants (Figure 4).

**Figure 5:**
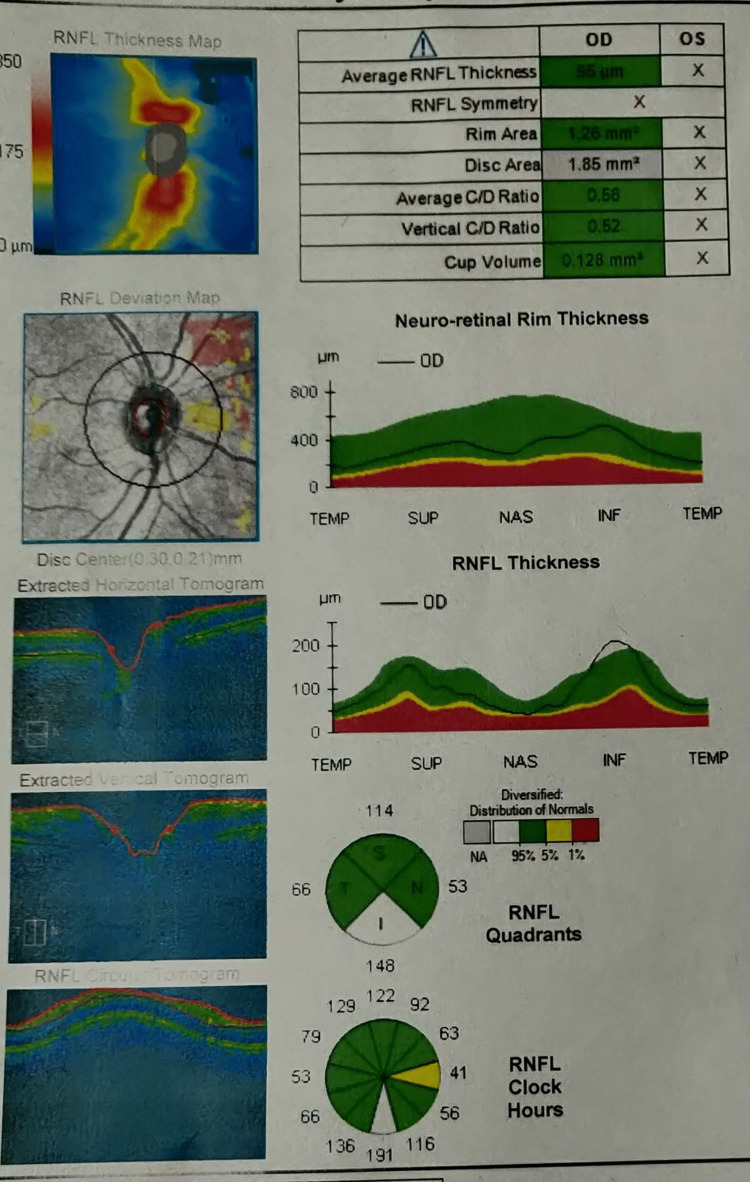
OCT ONH of the right eye showing normal thickness of peripapillary nerve fiber OCT ONH - optical coherence tomography of the optic nerve head

In the right eye, it was normal (Figure 5). The patient was advised prophylactic Nd:YAG laser peripheral iridotomy in both eyes.

## Discussion

Coloboma of the fundus is a rare anomaly that occurs in 0.5-2.2 of every 1,00,000 live births due to defective closure of the embryonal fissure [4]. Coloboma involving the disc or optic nerve head is said to be due to incomplete closure of the proximal part of the embryonal fissure during the sixth week of gestation [3]. Theories stating the existence of coloboma are impaired closure of the optic fissure, PAX-2 gene mutation leading to abnormal astrocytic differentiation, and defective migration of neural crest cells [5]. The prevalence of disc coloboma has been reported to be 0.14% in the general population [6].

It is uncommon to have an isolated optic nerve head coloboma without any systemic pathology. These can be sporadic, familial, or can be associated with genetic abnormalities. There are no racial or sexual predilections [7,8]. Our patient had isolated unilateral coloboma involving the disc without any other ocular anomaly or systemic association. As per Ida Mann's classification, there are seven types of fundal colobomas [9]. Type four is coloboma involving the disc, as seen in our case. Based on location and disc involvement, Gopal et al. classified colobomas into six types [10]. In types one to three, the fundal coloboma did not reach the optic disc, while in types four to six, the disc is enclosed within the coloboma. Congenital optic disc anomalies like coloboma involving disc or optic disc coloboma, morning glory syndrome, and optic disc pit are frequently misinterpreted as open-angle glaucomatous disc cupping [11,12]. As seen in glaucoma patients, coloboma involving disc or optic disc coloboma exhibits similar morphological appearance, visual field abnormalities on perimetry, and nerve fiber loss on optical coherence tomography.

We report a case of both eyes angle closure glaucoma, having normal optic disc with cup disc ratio of 0.3 without any peripapillary slit or wedge defects in right eye. The left eye showed coloboma involving the disc without any anomalous blood vessels. Optical coherence tomography in the right eye was normal, while the left eye showed decreased peripapillary nerve fiber thickness in the superior, inferior, and nasal quadrants. Controlling intraocular pressure and averting additional nerve fiber and ganglion cell loss are the primary goals of glaucoma treatment. Measurement of intraocular pressure, nerve fiber loss on OCT ONH, and visual field loss on perimetry are used to track how well glaucoma patients are responding to treatment. In our patient, in the left eye, only the temporal quadrant has normal thickness of the retinal fibre layer. In order to monitor retinal nerve fiber loss on OCT ONH, a further decrease in thickness in the superior, inferior and nasal quadrants, as well as a decrease in the temporal quadrant from present values, can be helpful. There will be a scotoma extending from the blind spot on visual field tests. Consequently, it will be challenging to track the disease progression in the left eye.

## Conclusions

Eyes with congenital disc coloboma are commonly misdiagnosed as open-angle glaucoma. As opposed, our patient is a case of angle closure glaucoma in both eyes with unilateral left eye coloboma involving the disc without any systemic abnormalities. Assessment of glaucomatous damage or progression of visual field loss is challenging in patients with disc coloboma and a known case of glaucoma.
